# Evaluation of celery extract as a natural alternative to sodium nitrite in fresh chicken sausages during refrigerated storage

**DOI:** 10.1002/jsfa.70329

**Published:** 2025-11-17

**Authors:** Ana Caroline Silvestre Barbosa Alessi, Nathália Letícia Hernandez Brito, Adriana Aparecida Droval Arcain, Flávia Aparecida Reitz Cardoso

**Affiliations:** ^1^ Postgraduate Program of Food Technology (PPGTA) Federal University of Technology – Paraná (UTFPR) Campo Mourão Brazil; ^2^ Postgraduate Program of Technological Innovations (PPGIT) Federal University of Technology – Paraná Campo Mourão Brazil

**Keywords:** meat preservation, natural curing agents, lipid oxidation, microbial safety, meat color stability

## Abstract

**BACKGROUND:**

Fresh chicken sausages are highly perishable and require preservation strategies to ensure quality and safety. Sodium nitrite is traditionally used to inhibit microbial growth and stabilize color; however, concerns regarding the formation of carcinogenic *N*‐nitroso compounds have increased interest in natural alternatives. Celery extract, rich in natural nitrite and bioactive compounds, exhibits antioxidant and antimicrobial properties, making it a promising substitute.

**RESULTS:**

Sausages were prepared with celery extract, sodium nitrite, or no additive (control) and stored at 5 °C for 14 days. Physicochemical parameters (pH, moisture loss, cooking weight loss, water‐holding capacity, and lipid oxidation) and color parameters (L*, a*, b*, and Δ*E*) were analyzed on days 1, 7, and 14. Two‐way analysis of variance and principal component analysis revealed significant interactions between treatment and time (*P* < 0.05). Celery extract‐treated sausages showed higher initial redness (a*) but greater total color change (Δ*E* = 8.56) than sodium nitrite (Δ*E* = 3.11). Lipid oxidation values ranged from 0.79 mg malondialdehyde (MDA) kg^−1^ (control, day 14) to 2.62 mg MDA kg^−1^ (nitrite, day 1). Microbiological analyses confirmed the absence of *Salmonella* spp., *Escherichia coli*, and coliforms in all treatments.

**CONCLUSION:**

Celery extract provided good initial color stabilization and moderate antioxidant activity, performing similarly to sodium nitrite in several physicochemical parameters. However, greater color variation and limited oxidative stability over time highlight the need for formulation optimization or combination with other natural antioxidants. Celery extract is a promising clean‐label alternative to nitrite; however, further studies are needed on residual nitrite content, sensory acceptance, and extended shelf life. © 2025 The Author(s). *Journal of the Science of Food and Agriculture* published by John Wiley & Sons Ltd on behalf of Society of Chemical Industry.

## INTRODUCTION

Food safety and nutrition guidelines define food as any substance or mixture in solid, liquid, or other suitable forms intended to provide essential nutrients for human growth, maintenance, and development. Due to their biological activity, all foods are susceptible to contamination and deterioration by enzymes and microorganisms, making preservation crucial.^1^

Although not essential in the human diet, meat is valued for its high‐quality proteins, vitamins, minerals, fatty acids, and trace elements.^2^, ^3^ Historically, salting, smoking, and fat preservation techniques were developed and refined over time.^4^ With the advancement of food technology in the 20th century, preservation techniques evolved, industrializing meat processing and extending shelf life.^5^

Fresh sausages, a significant segment of processed meats, are particularly susceptible to microbial growth and lipid oxidation due to their high water content and nutrient composition. Consequently, preservatives such as nitrites and nitrates are commonly used to inhibit bacterial growth, enhance color stability, and improve sensory attributes.^6^, ^7^ However, concerns about the potential carcinogenic effects of *N*‐nitroso compounds formed by nitrites have led to a growing demand for natural alternatives.^8^, ^9^

Plant‐based extracts have been studied as potential replacements for synthetic nitrites in meat products, with celery extract emerging as a viable alternative due to its naturally high nitrite content and bioactive compounds.^10^ Celery extract exhibits antioxidant, antimicrobial, and color‐enhancing properties, making it a promising ingredient in processed meat formulations.^11^ Additionally, it contains iron, sodium chloride, volatile sulfur compounds, and antioxidant vitamins,^12^, ^13^ further supporting its potential as a functional ingredient.

Several studies have explored the effectiveness of celery extract in preserving the quality of processed meats. Barbosa *et al*.^10^ highlighted its capacity to delay lipid oxidation, while Ramachandraiah and Chin^14^ demonstrated its potential to replace synthetic additives in pork sausages. Lee *et al*.^15^ reported its antimicrobial properties and ability to enhance sensory attributes such as juiciness and texture. Additionally, Pennisi *et al*.^16^ found that celery extract effectively replaced nitrite in mixed fresh sausages, particularly when combined with rosemary extract.

Despite extensive research on celery extract in processed meats, a gap remains in the literature regarding its application in fresh chicken sausages. While previous studies have focused mainly on pork and beef products, the impact of celery extract on lipid oxidation, color stability, and microbial safety in fresh poultry sausages remains underexplored. Since nitrite is important in preventing pathogenic bacterial growth and maintaining meat color, further investigation is warranted to determine whether celery extract can provide comparable benefits without compromising food safety.

This study aims to evaluate the potential of celery extract as a natural alternative to sodium nitrite in fresh chicken sausages, focusing on its effectiveness in maintaining meat color, delaying lipid oxidation, and ensuring microbiological safety during a 14‐day refrigerated storage period. Addressing these critical aspects, the research seeks to contribute to the ongoing search for safer, more natural preservation methods in processed meats while ensuring quality and food safety.

## MATERIALS AND METHODS

### Ingredients and sample preparation

Fresh chicken sausages were prepared using thigh fillets, chosen for their natural fat content, eliminating the need for additional fat sources. The seasonings included salt, garlic powder, ground white pepper, monosodium glutamate, smoked paprika, and dried fine herbs sourced from a local market. Additionally, celery extract, curing salt (LF 604, composed of 90% sodium chloride and 10% sodium nitrite), and sodium erythorbate were incorporated as curing and antioxidant agents. The celery extract used was a liposoluble oleoresin, obtained from celery seeds, provided by NewMax Industrial (Americana, Brazil). According to the supplier's certificate, the oleoresin presented a total lipid content of approximately 88.0 g 100 g^−1^. No solvents or additional carriers were declared in its composition.

The chicken meat and skin were chilled at 4 °C for 24 h prior to processing. Subsequently, they were ground using a manual grinder (Malta, meat grinder No. 10, Malta Innovative Industry in Equipment and Household Utilities, Caxias do Sul, Brazil) with a 6 mm disc. Chilled water and seasonings were added to the mixture, followed by thorough manual mixing for 10 min to ensure uniform distribution of ingredients.^17^ Proper sanitation procedures were followed before and after mixing to avoid cross‐contamination. The homogenized mixture was then divided into three formulations, as shown in Table 1.

**Table 1:** Ingredients and proportions used for obtaining C, N, and E formulations

Ingredient	C	N	E
Water	6%	6%	6%
Salt	2%	1.75%	2%
Garlic powder	0.20%	0.20%	0.20%
Ground white pepper	0.04%	0.04%	0.04%
Monosodium glutamate	0.20%	0.20%	0.20%
Dehydrated fine herbs	0.04%	0.04%	0.04%
Smoked paprika	1%	1%	1%
Sodium erythorbate	0.25%	0.25%	0.25%
LF 604 curing salt (NaCl + NaNO_2_)	—	0.25%	—
Celery extract	—	—	0.002%

Sample C does not contain added curing salt; sample N contains sodium nitrite; sample E contains celery extract.

To adjust for the sodium chloride content in the nitrite‐added formulation, the amount of sodium chloride was recalculated to compensate for the sodium chloride present in the curing salt.^18^ Since no official regulation specifies the appropriate proportion of celery extract for nitrite substitution, the concentration was based on the limits for carotenoid usage as defined in RDC 272.^19^

### Stuffing and storage conditions

After formulation, the mixtures were stored at 5 °C for 6 h to allow for binding and curing. Subsequently, the sausages were manually stuffed into natural sheep casings using a manual sausage stuffer (Lemaq, Diadema, Brazil). The casings were sourced commercially from local Brazilian suppliers (Campo Mourão, Paraná, Brazil), having a diameter ranging between 20 and 22 mm and lengths cut into approximately 15 cm segments. The sausages were placed in polyethylene vacuum‐sealed bags (Cryovac P6000, Sealed Air, São Paulo, Brazil) with low oxygen permeability (<10 cm^3^ m^−2^ d^−1^ at 23 °C) and stored at 5 °C (Panasonic, São Paulo, Brazil) for 24 h before analysis. All utensils were thoroughly cleaned and sanitized between different formulations to prevent cross‐contamination.^20^

### Microbiological analysis

The following microbiological analyses were performed to assess the microbiological quality of the chicken sausages.

#### Total viable count

The total viable count (TVC) was determined using the pour plate method.^21^ A 1 g sample from each formulation was homogenized in 9 mL peptone water and serially diluted. Aliquots (0.1 mL) of appropriate dilutions were plated onto Plate Count Agar (Merck, Darmstadt, Germany) and incubated at 30 °C for 48 h. The results were expressed as colony‐forming units per gram (CFU g^−1^) of the sample.

#### Coliforms (*Escherichia coli* and total coliforms)

Coliforms were enumerated using the most probable number (MPN) method as described in the Brazilian Microbiological Standards.^22^ For total coliforms, the samples were diluted and plated on MacConkey agar for *E. coli* and Violet Red Bile Glucose Agar (VRBGA; Merck). Plates were incubated at 37 °C for 24 h, and results were expressed as MPN g^−1^ of the sample.

#### *Salmonella* spp.

*Salmonella* was detected by the enrichment method, followed by selective plating.^23^ A 25 g sample was enriched in buffered peptone water and incubated at 37 °C for 24 h. After enrichment, 0.1 mL of the broth was streaked onto xylose lysine deoxycholate agar plates, which were incubated at 37 °C for 24–48 h. Colonies with typical *Salmonella* morphology were confirmed using biochemical tests.

### Physicochemical analyses

#### pH determination

The pH of the samples was measured in triplicate using a portable pH meter (HI 99163, Hanna Instruments, Woonsocket, RI, USA) with a penetration electrode, following the methodology described by Olivo *et al*.^17^ The electrode was inserted into each sausage sample's points (left, right, and center) to ensure accurate measurement.

#### Water‐holding capacity

The water‐holding capacity (WHC) was determined following the method of Silva *et al*.^24^ A 5 g sample was placed between two pieces of quality qualitative filter paper (12.5 cm diameter, Grade 1 (Whatman, Maidstone, UK)) and pressed with a 10 kg cylindrical weight for 10 min in a desiccator saturated with potassium chloride (KCl). The weight difference before and after pressing was used to calculate WHC.

#### Cooking weight loss

Cooking weight loss (CWL) was evaluated according to Sebranek *et al*.^18^ The sausages were weighed (Bioscale, São Paulo, Brazil), cooked in an air fryer (Britânia Eletrodomésticos S.A., Curitiba, Brazil) at 200 °C for 12 min until an internal temperature of 80 °C was reached, then cooled to 40 °C and reweighed. The percentage of weight loss was calculated.

#### Objective color determination

Objective color (L*, a*, b*) was measured using a colorimeter (Delta Color, São Leopoldo, Vista, Brazil), following the CIELAB color space system.^25^ The readings were performed in triplicate at three points on the sausage surface to ensure consistency. Additionally, the total color difference (Δ*E*) was calculated to better represent overall color changes during storage, considering the combined variations in L*, a*, and b*. The calculation was performed according to Eqn (1):

(1) $$∆E=\sqrt{{\left(∆L\right)}^{2}+{\left(∆a\right)}^{2}+{\left(∆b\right)}^{2}}$$

where L*, a*, and b* are color parameters at day 0 and day *t*.

#### Moisture loss

Moisture content was determined using the desiccation method, following the official procedure described by the Adolfo Lutz Institute.^26^ A 5 g sample was dried at 105 °C for 3 h in an air‐circulating oven, or until constant weight was achieved, which proved sufficient due to the small sample size. After drying, samples were cooled in a desiccator for 2 h before final weighing.

#### Lipid oxidation (TBARS analysis)

Lipid oxidation was measured using the thiobarbituric acid reactive substances (TBARS) method, as described by Queiroz and Silva^27^ and Selani *et al*.^28^ TBARS values were determined spectrophotometrically (ultraviolet–visible, UV‐1800, Shimadzu, Kyoto, Japan) at 538 nm, using a standard MDA curve, and expressed as mg MDA kg^−1^ of sample.

Two solutions were prepared for TBARS analysis in sausages: 2‐thiobarbituric acid (TBA) and the other containing trichloroacetic acid (TCA), propyl gallate, and ethylenediaminetetraacetic acid (EDTA). Samples (5 g each) were weighed in triplicate in a beaker, and 25 mL of the second solution (TCA 7.5%, propyl gallate 0.1%, EDTA 0.1%) was added to each. After homogenization (713D, Fisatom, São Paulo, Brazil), the mixture was filtered through a glass funnel using Qualy qualitative filter paper (12.5 cm diameter).

Five milliliters of filtrate was transferred to a test tube, and 5 mL TBA solution was added. This process was repeated for all samples, and the test tubes were capped and placed in a water bath (837–2, WEA Laboratory Supplies and Services Ltda., Hortolândia, Brazil) at 100 °C for 40 min to form the colored complex.

A control was performed by adding 5 mL TCA solution and 5 mL TBA solution to a test tube and subjecting it to the same water bath conditions to adjust the spectrophotometer. All sample readings were taken at a wavelength of 538 nm using a UV–visible spectrophotometer, and the results were expressed as mg MDA kg^−1^ of meat.

### Statistical analysis

Data were statistically analyzed using two‐way analysis of variance (ANOVA) to evaluate the effects of formulation (control, sodium nitrite, celery extract), storage time (days 1, 7, and 14), and their interaction on the physicochemical properties. Post hoc comparisons were conducted using Tukey's test at a significance level of 5% (*P* < 0.05) using Statistica 12.0 software (StatSoft, Tulsa, OK, USA). Principal component analysis (PCA) was conducted for physical–chemical and lipid oxidation data using OriginPro 2020b (OriginLab Corporation, Northampton, MA, USA) to assess clustering and variability among formulations.

## RESULTS AND DISCUSSION

The quality of fresh meat products, such as fresh sausages, is influenced by various factors, including physicochemical characteristics such as pH, CWL, moisture, WHC, and lipid oxidation. These parameters play important roles in the acceptability and shelf life of the product. Therefore, monitoring and understanding the changes in these properties over time during storage are essential.

Additionally, the microbiological quality of the product is equally important, as the presence of pathogenic microorganisms can compromise the safety and sensory properties of the sausage. The study analyzed the mean values and standard deviations of pH, moisture, CWL, WHC, objective color, lipid oxidation, and microbiological parameters such as TVC, *E. coli*, total coliforms, and *Salmonella* spp. in fresh sausage formulations, explicitly evaluating the storage intervals of 1, 7, and 14 days.

The results obtained were subjected to ANOVA and Tukey's test (*P* < 0.05), providing a deeper understanding of the changes in these properties during storage, as well as the microbiological stability of the product. The incorporation of various curing agents and antioxidants, such as sodium nitrite and celery extract, was evaluated for their impact on the physicochemical characteristics and microbiological safety of the sausages.

### Microbiological analysis

Table 2 presents the results of the microbiological analyses. The TVC was satisfactory, with all treatments showing values below 3.0 × 10^2^ CFU g^−1^. These values are well below the recommended safety limit of 10^3^ CFU g^−1^, indicating that the product has good microbiological quality. No significant differences in microbial growth were observed between the treatments with nitrite and celery extract, suggesting that neither additive negatively affected the natural microbiota of the chicken meat during the manufacturing and storage process.

**Table 2:** Microbiological results of chicken sausages

Microbiological parameter	C	N	E	Safety limit (RDC 12/2001, ANVISA)
TVC (CFU g^−1^)	2.5 × 10^2^	1.8 × 10^2^	3.0 × 10^2^	<10^3^
*Escherichia coli* (MPN g^−1^)	< 3	< 3	< 3	<3
Total coliforms (MPN g^−1^)	< 3	< 3	< 3	<10^3^
*Salmonella* spp.	Absent	Absent	Absent	Absent

Sample C does not contain added curing salt; sample N contains sodium nitrite; sample E contains celery extract. ANVISA, Brazilian Health Regulatory Agency; CFU, colony‐forming units; TVC, total viable count; MPN, most probable number.

The absence of *E. coli* in all formulations (control, nitrite, and celery extract) indicates that the manufacturing process followed good hygiene practices, ensuring that the risk of fecal contamination was adequately controlled. *Escherichia coli* could suggest failures in sanitary controls, and its absence is crucial to ensure food safety.

The coliform count also remained below the safety limit of 10^3^ MPN g^−1^, with all treatments showing values lower than 3 MPN g^−1^. The absence of coliforms is a positive indication that hygiene control measures during production and storage were effective. Coliforms are commonly used as indicators of inadequate hygiene and handling conditions, but the results indicate that the products meet the expected microbiological standards for processed meats.

The absence of *Salmonella* in all samples is a particularly notable result, as *Salmonella* is a highly virulent pathogen responsible for causing severe food poisoning. Its absence in all treatments strongly indicates that the manufacturing process effectively prevented cross‐contamination and that the storage and processing conditions were appropriate.

The microbiological results for the chicken sausages, whether with nitrite, celery extract, or the control, were satisfactory, indicating that all products comply with the food safety standards established by health authorities. The microbiological quality of the sausages was preserved in all formulations, with no presence of pathogenic contaminants, including *E. coli*, *Salmonella* spp., and coliforms, and the TVC was within the recommended limits. These results suggest that using nitrite and celery extract as a nitrite substitute did not compromise the product's safety, with both being effective in preserving the microbiological quality of the chicken sausages.

### Physicochemical analyses

To evaluate the physicochemical properties of the fresh sausage formulations over time, Table 3 presents the pH, moisture loss, CWL, WHC, and lipid oxidation values on days 1, 7, and 14 of storage. These parameters are critical for understanding the product's quality and stability, as they directly influence the sausages’ texture, flavor, and shelf life. The following analyses provide insights into how the formulations, incorporating different curing agents and antioxidants, respond to storage conditions over the specified period.

**Table 3:** pH, moisture loss, cooking weight loss, water‐holding capacity, and lipid oxidation values of fresh sausage formulations on days 1, 7, and 14

Sample	Time (days)		
	1^a^	7	14
pH			
C	6.32bA ± 0.01	6.27bB ± 0.01	6.16bC ± 0.05
N	6.35bAB ± 0.03	6.38aA ± 0.01	6.33aB ± 0.01
E	6.40aA ± 0.01	6.35aB ± 0.01	6.21abC ± 0.02
*Moisture loss* (%)			
C	38.29bA ± 1.45	39.70aA ± 0.82	40.12aA ± 1.76
N	50.07aA ± 2.41	38.44aB ± 1.24	39.47aB ± 2.14
E	47.33aA ± 1.29	37.69aB ± 1.04	38.14aB ± 0.32
*Cooking weight loss* (%)			
C	13.52aB ± 0.61	14.91aB ± 0.61	16.25aA ± 0.33
N	16.09aA ± 1.09	14.39aB ± 0.21	18.39aA ± 2.50
E	12.77aB ± 0.66	11.53bB ± 0.51	18.00aA ± 0.08
*Water‐holding capacity* (%)			
C	89.24aA ± 0.98	89.68aA ± 1.36	86.83aA ± 1.44
N	88.91aA ± 1.61	88.91aA ± 1.57	89.43aA ± 0.60
E	86.57aA ± 1.20	87.81aA ± 0.73	87.13aA ± 2.26
*Lipid oxidation* (mg MDA kg^−1^)			
C	1.05bB ± 0.13	2.48aA ± 0.50	0.79bB ± 0.18
N	2.62aA ± 0.59	1.81abB ± 0.96	1.67aB ± 0.53
E	1.62abA ± 0.95	0.83bB ± 0.23	1.84aA ± 0.63

Means in the same column, followed by different lower‐case letters, differ using the Tukey test at a significance level of 5% between the samples at each of the different times analyzed. Means in the same line, followed by different upper‐case letters, differ from each other using the Tukey test at a significance level of 5% about each of the samples at times 1, 7, and 14 days. Sample C does not contain added curing salt; sample N contains sodium nitrite; sample E contains celery extract. MDA, malondialdehyde.

^a^ Day 1 refers to 24 h after processing.

A two‐way ANOVA revealed significant interactions between treatment (control, sodium nitrite, celery extract) and storage time (days 1, 7, and 14) for the physicochemical parameters evaluated (*P* < 0.05). These findings indicate that the impact of each treatment on the quality characteristics of fresh sausages significantly depends on the storage period, as detailed in Tables 3 and 4.

**Table 4:** Color parameters for fresh sausage formulations on days 1, 7, and 14

Sample	Time (days)		
	1^a^	7	14
L*			
C	42.86aB ± 0.78	48.26aA ± 2.85	47.29aA ± 1.44
N	43.49aA ± 0.16	45.30aA ± 0.22	43.83aA ± 0.60
E	45.20aA ± 0.83	44.55aA ± 0.93	44.36aA ± 2.26
a*			
C	15.80bA ± 0.48	14.69bA ± 1.26	13.20bA ± 1.05
N	18.62aA ± 0.73	18.08aA ± 0.25	15.53aB ± 0.80
E	19.31aA ± 0.42	17.98aA ± 0.39	13.34bB ± 0.25
b*			
C	21.96cB ± 0.78	27.50aA ± 0.67	20.96aB ± 1.46
N	24.89bB ± 0.67	27.93aA ± 0.45	24.98aB ± 0.86
E	29.53aA ± 0.27	27.67aA ± 0.67	23.46aB ± 1.68
∆E (1–14 days)			
C	5.23		
N	3.11		
E	8.56		

Means in the same column, followed by different superscript lowercase letters, differ using the Tukey test at a significance level of 5% between the samples at each of the different times analyzed. Means in the same row, followed by different upper‐case letters, differ from each other using the Tukey test at a significance level of 5% about each of the samples at times 1, 7, and 14 days. Sample C does not contain added curing salt; sample N contains sodium nitrite; sample E contains celery extract.

^a^ Day 1 refers to 24 h after processing.

The pH values ranged from 6.16 ± 0.05 (control sample on day 14) to 6.40 ± 0.01 (sample with celery extract on day 1). A more pronounced decrease in pH was observed over the 14 days for the control (C) and celery extract (E) samples. A significant interaction (treatment × time) was observed for pH (*P* < 0.05), indicating that the variation in pH during storage depended on the type of preservative used. The reduction in pH in fresh meat products is often associated with microbial activity and the degradation of protein components.^29^ Previous studies have also reported a reduction in pH in fresh sausages containing natural extracts, as observed by Siqueira,^30^ where the pH varied from 6.18 to 6.09 during storage, a finding consistent with the values reported in the present study.

Regarding moisture, the loss remained consistent over time for the control sample, whereas the samples with nitrite (N) and celery extract (E) exhibited a reduction in moisture loss over the 14‐day period. The significant interaction observed for moisture loss (*P* < 0.05) indicates that the water retention capacity of the formulations varied distinctly during storage, depending on the preservative used. This may be related to the ability of plant extracts to retain water and form a gel matrix that helps minimize moisture loss.^31^

CWL increased for all samples between days 1 and 14, which can be attributed to the degradation of myofibrillar proteins, thereby affecting water retention.^32^ A significant interaction (treatment × time) was observed for CWL (*P* < 0.05), indicating that the treatments had variable impacts on this parameter throughout storage. There were no significant differences in WHC during the period, although a slight reduction was observed for the control sample. This behavior may be explained by the reduction in pH, which negatively influences WHC.^33^

Regarding lipid oxidation, the highest value observed was 2.62 ± 0.59 mg MDA kg^−1^ in the nitrite sample on day 1 (24 h post‐processing), while the lowest value was 0.79 ± 0.18 mg MDA kg^−1^ in the control sample on day 14. A significant interaction (treatment × time) was observed for lipid oxidation (TBARS) (*P* < 0.05), indicating that the antioxidant effectiveness of the treatments varied throughout the storage period. There was a reduction in lipid oxidation for the nitrite sample over time and an increase for the celery extract sample. These results are consistent with the literature, which suggests that celery extract, due to its antioxidant content, may delay lipid oxidation compared to nitrite, but not with the same efficacy.^34^ However, the observed increase in oxidation for the celery extract sample may indicate the need for higher concentrations or a combination with other natural antioxidants to improve lipid stability.^35^

### Color analysis

The results for color parameters (L*, a*, and b*) are presented in Table 4. Lightness (L*) remained consistent for all samples over time. However, the a* parameter (red intensity) decreased over time, with the highest values observed in the celery extract sample, which may be attributed to the presence of phenolic compounds that interact with meat pigments, intensifying the red color, as described by Honikel.^32^ Similarly, the b* parameter (yellow intensity) decreased over time for the celery extract sample, indicating that the characteristic color of celery extract did not negatively interfere with the sausage's typical pink color formation. A significant interaction between treatment and time was observed for color parameters (*P* < 0.05), indicating that the impact of preservatives on color stability throughout storage depends on the type of formulation used.

Previous studies, such as that by Sebranek *et al*.,^18^ confirm that celery extract can effectively maintain color in cured meat products. However, efficacy may vary depending on concentration and processing conditions. The ability of celery extract to preserve the red color may be related to the formation of stable complexes with myoglobin, thereby preventing oxidation and discoloration, as Pegg and Shahidi^36^ suggested.

To complement the colorimetric evaluation, the total color difference (Δ*E*) between day 1 and day 14 was also calculated for each formulation. The sample treated with celery extract (E) exhibited the highest Δ*E* value (8.56), indicating a more noticeable color change over the storage period, despite its initially high a* values. The control sample (C) presented an intermediate Δ*E* value (5.23), reflecting moderate color degradation in the absence of stabilizing agents. In contrast, the sample containing sodium nitrite (N) showed the lowest Δ*E* (3.11), suggesting greater color stability throughout storage. These results reinforce the effectiveness of synthetic nitrite in preserving the color of cured meat products and suggest that, although celery extract is a promising natural alternative, it may require formulation optimization or combination with other natural antioxidants to maintain visual quality over more extended storage periods.

**Figure 1:**
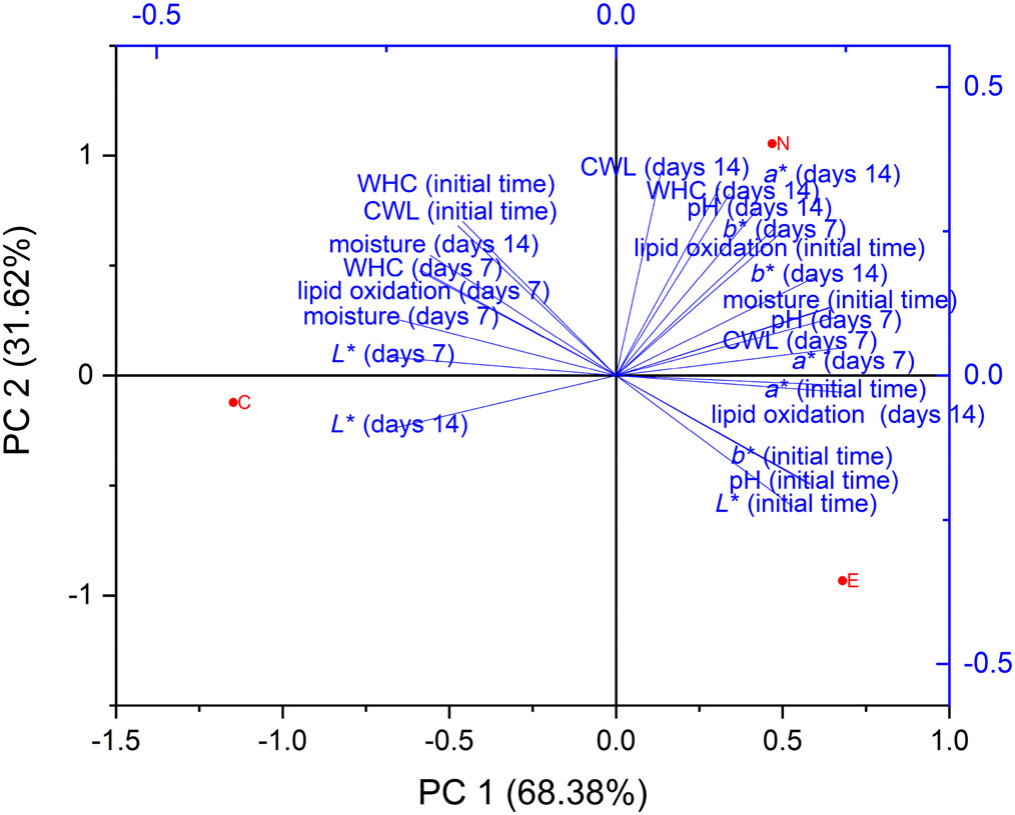
Biplot of principal component analysis for physicochemical analyses of fresh sausage samples over the 14‐day analysis period. WHC, water‐holding capacity; CWL, cooking weight loss. Sample C does not contain added curing salt; sample N contains sodium nitrite; sample E contains celery extract.

PCA analysis (Fig. 1) revealed differences in the physicochemical characteristics of sausage formulations. The celery extract‐treated sample (E) exhibited higher initial values of redness (a*) and lipid oxidation at day 14, indicating that, despite initial color stabilization, celery extract may not effectively control lipid oxidation throughout the entire storage period. Conversely, sodium nitrite‐treated samples (N) exhibited more stable redness values throughout storage, reinforcing nitrite's known efficiency for maintaining meat color.^32^ The control sample (C), without additives, clearly demonstrated higher susceptibility to oxidative deterioration. These results underscore the importance of selecting preservatives based on specific desired outcomes, as natural antioxidants may provide initial protection but may require further optimization for long‐term storage.

In the quadrant where the E sample is located, we observe an association with variables such as initial pH, a*, b*, and lipid oxidation on the 14th day. These results indicate that celery extract has a positive effect on color stability and reduces lipid oxidation over time, which is consistent with studies by Alahakoon *et al*.,^37^ demonstrating that natural compounds, such as celery extract, possess antioxidant activities that can preserve color and delay oxidative deterioration in meat products.

On the other hand, the N sample, located in the upper right quadrant, correlates with variables such as CWL on days 1 and 14, a* on the 14th day, and lipid oxidation on the first day. This suggests that nitrite has an immediate impact on color preservation and the inhibition of initial lipid oxidation, as demonstrated by studies such as that by Honikel,^32^ which highlights nitrite as one of the most effective preservatives in meat products due to its ability to form nitroso‐myoglobin, stabilizing the red color and inhibiting bacterial growth.

The control sample (C), positioned in the lower left quadrant, exhibits lower color stability and higher lipid oxidation, particularly on day 7, as indicated by the L* and moisture variables on days 7 and 14. These results confirm that, without the addition of preservatives, fresh sausages tend to deteriorate more quickly, as observed by Sebranek *et al*.,^38^ who reported that the absence of nitrite or natural antioxidants led to a significant loss of quality, particularly in terms of color and oxidative stability.

**Figure 2:**
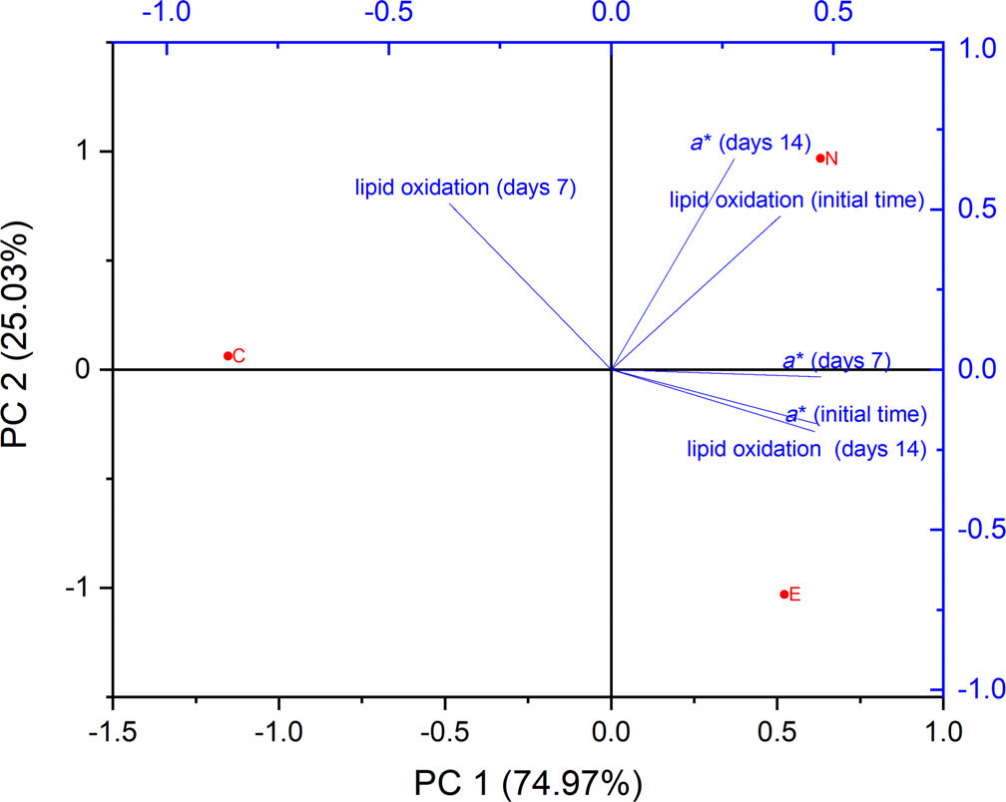
Biplot of principal component analysis for lipid oxidation and a* color parameter in fresh sausage samples over the 14‐day analysis period. Sample C does not contain added curing salt; sample N contains sodium nitrite; sample E contains celery extract.

The second PCA focused solely on lipid oxidation and the a* color parameter to investigate the relationship between these two factors over the storage period. These variables were chosen because lipid oxidation is a critical process contributing to the deterioration of meat products, directly affecting flavor, aroma, and overall acceptability (Fig. 2). Conversely, the a* color parameter, representing redness intensity, is important for the appearance and perception of freshness in sausages. Since color is a key visual indicator of quality for consumers, understanding how oxidation affects this characteristic is essential for evaluating the effectiveness of preservatives, such as celery extract and nitrite, in maintaining color stability during storage.

Based on the correlation analysis between lipid oxidation and the a* color parameter over 14 days, PCA revealed that PC1 explained 74.97% of the variance, while PC2 accounted for 25.03%. This high percentage of variance captured by PC1 suggests a strong relationship between lipid oxidation and color stability in fresh sausages. In the first component (PC1), there is a positive correlation between lipid oxidation on day 14 and the a* color parameter on days 1 and 7, indicating that the sample treated with celery extract (E) exhibited the highest lipid oxidation by the 14th day and the highest a* values in the initial stages. This finding is consistent with studies reporting that natural antioxidants can enhance color stability but may be less effective in preventing long‐term lipid oxidation than synthetic additives, such as nitrite.

In contrast, the second component (PC2) shows a positive correlation between lipid oxidation on days 1 and 7 and the a* color parameter on day 14. This indicates that the control sample (C) had the highest lipid oxidation on day 7, confirming the susceptibility of untreated sausages to oxidative degradation. Meanwhile, the nitrite‐added sample (N) showed the highest lipid oxidation on day 1 but maintained a higher a* value on day 14. This result supports observations of nitrite's effectiveness in maintaining color stability, even in the presence of early oxidation. The positioning of the samples highlights the distinct roles of preservatives in maintaining fresh sausage quality: celery extract (E) contributed to initial color stability and reduced lipid oxidation in later stages, whereas nitrite (N) was more effective in long‐term color preservation, despite early oxidation. The control sample (C) demonstrated the disadvantages of not using any preservative, resulting in higher oxidation and a less stable color over time. These findings underscore the importance of selecting suitable preservatives based on the desired shelf life and quality attributes of the fresh sausage. While natural extracts, such as celery, may appeal to consumers seeking clean‐label products, their efficacy compared to synthetic additives like nitrite may require careful consideration, depending on specific quality goals.

Thus, celery extract appears to be a promising substitute for sodium nitrite in fresh chicken sausage formulations, based on the analyses conducted in this study, as its behavior was not significantly different from that of synthetic curing salt and showed better performance than the control sample. However, it is essential to note that further studies, particularly microbiological analyses, are needed to confirm the efficacy of this potential substitute, as sodium nitrite's primary utility is to prevent the growth of pathogenic microorganisms. Additionally, sensory analysis should be conducted to validate the quality and acceptance of the formulation with celery extract. Plant extracts used to replace nitrite can often affect the flavor of meat products, making sensory evaluation and the determination of volatile flavor compounds particularly important. Further studies are also recommended to understand the behavior of celery extract in fresh chicken sausage over a more extended storage period, particularly regarding lipid oxidation, and to identify the components present in celery extract.

### Study limitations and recommendations for future research

One significant limitation of this study is the absence of residual nitrite analysis in sausages treated with celery extract. While natural sources of nitrite, such as celery extract, are widely considered safer by consumers, a critical misconception persists regarding their safety profile. Natural sources of nitrite can also accumulate and generate residual nitrite, potentially leading to the formation of harmful *N*‐nitroso compounds when consumed in excess. Studies have highlighted that celery‐derived nitrite may vary significantly depending on the extraction method and source, posing challenges for standardization.^18^, ^34^ Thus, future research should address residual nitrite quantification explicitly and focus on the dose‐dependent safety and effectiveness of celery extract as a natural curing agent. Additionally, sensory studies to assess consumer acceptability and the influence of natural nitrite sources on flavor should also be included to provide a comprehensive understanding of the feasibility of natural alternatives.

## CONCLUSIONS

The use of powdered celery extract in fresh chicken sausages showed promising initial effects in maintaining desirable color and reducing moisture loss compared to the control. Celery extract‐treated samples exhibited higher redness (a*) and moderate antimicrobial and antioxidant activities. Based on this study, it was possible to verify that celery extract behaved similarly to sodium nitrite in all the parameters analyzed. Regarding pH, there was considerable similarity among the three samples. In terms of moisture, both the nitrite and celery extract formulations exhibited lower losses than the control over time.

For CWL and WHC, both N and E samples showed increased CWL values and maintained relatively stable WHC, unlike the control, which exhibited a decrease in both CWL and WHC. Regarding lipid oxidation, the nitrite‐treated sample presented higher MDA values on day 1, whereas the control showed the lowest levels on day 14. It was also noted that the inclusion of chicken skin may have influenced lipid oxidation behavior. Nonetheless, all samples remained within acceptable limits for consumption throughout storage.

Color evaluation revealed that all samples experienced some degradation due to protein denaturation. The control sample showed reductions in L*, a*, and b*, while the N and E samples maintained L* values more consistently. The E sample, however, showed a significant drop in b* and changes in a* that reflected progressive myoglobin oxidation – a reaction that contributes to the desired cured color.

Two‐way ANOVA confirmed significant treatment × time interactions, demonstrating that preservative efficacy is influenced by storage duration. Although celery extract initially performed well, it exhibited a greater total color change (Δ*E* = 8.56) and limited oxidative control compared to sodium nitrite (Δ*E* = 3.11). Therefore, further studies are recommended to optimize the concentration of celery extract, explore synergistic combinations with other natural antioxidants, confirm its microbiological safety over extended storage periods, and conduct sensory evaluations to assess consumer acceptance.

## Data Availability

Data sharing not applicable to this article as no datasets were generated or analysed during the current study.
